# Perceived Stigma Among Adults With Alopecia Areata in the United States

**DOI:** 10.1111/1346-8138.17786

**Published:** 2025-05-23

**Authors:** Genevieve Gauthier, Kent A. Hanson, Helen Tran, Lynne Napatalung, Griffith Bell, Mojgan Sadrarhami, Lulu Lee, Nikoletta Sternbach, Ernest H. Law, Samantha K. Kurosky

**Affiliations:** ^1^ Pfizer Inc New York New York USA; ^2^ Department of Pharmacy Systems Outcomes and Policy, University of Illinois Chicago Chicago Illinois USA; ^3^ Icahn School of Medicine at Mount Sinai New York New York USA; ^4^ Oracle Life Sciences Austin Texas USA

**Keywords:** Alopecia areata, comorbidities, mental health, quality of life, stigma

## Abstract

Alopecia areata (AA) can significantly impact mental/emotional quality of life, which may be partially attributed to disease‐related stigma. We assessed patient‐reported stigma in US adults with AA using data collected from the 2023 US National Health and Wellness Survey. Demographics, clinical characteristics, comorbidities, and perceived stigma were analyzed for patients with physician‐diagnosed AA, stratified by self‐reported disease severity. Data were weighted to match demographics of the US adult population. Of 75 007 (weighted *n* = 255.7 million) adults who completed the survey, 859 (weighted *n* = 2.9 million; 1.1%) self‐reported physician‐diagnosed AA. Among those reporting physician‐diagnosed AA, 58.8%, 26.2%, and 15.0% self‐reported mild, moderate, and severe AA, respectively. Overall, 40.1% reported experiencing symptoms of diagnosed emotional/mental health conditions in the past 12 months; 10.8% were receiving psychological counseling, and 10.8% were receiving medications for AA‐related anxiety, depression, or sleep disorders. Internal or external stigma was reported by 79.2% of respondents. Adults with AA reported substantial emotional/mental health comorbidities, with few receiving counseling or therapeutic intervention. Dissatisfaction with their current hair growth was prevalent, and considerable stigma was reported across all levels of AA disease severity, highlighting the substantial impact of external perceptions of AA. Experiencing AA stigma may lead to social withdrawal and isolation, further exacerbating the psychosocial burden of AA.

## 1 Introduction

Alopecia areata (AA) is an autoimmune disease characterized by patchy or complete nonscarring hair loss on the scalp, with or without additional loss of facial and/or body hair [1]. Hair loss from AA is unpredictable. Some patients have multiple hair loss and regrowth episodes; others have persistent and widespread areas of hair loss [1].

AA can negatively impact quality of life [2]. Individuals report AA‐related stigma stemming from the presence of visible hair loss or feeling/looking vulnerable, which increases the likelihood of mental health comorbidities [3, 4]. Increased emotional burden and negative self‐perceptions related to AA can worsen symptoms of anxiety and depression [5, 6]. This study examined the prevalence of self‐reported psychosocial comorbidities and perceptions of AA‐related stigma in a generalizable sample of adults with AA in the US.

## 2 Methods

### 2.1 Study Design

This study used data collected in 2023 from the US National Health and Wellness Survey (NHWS), an annual, cross‐sectional, syndicated, internet‐based survey conducted among adults (≥ 18 years) between May and August 2023 [7]. The survey collects patient self‐reported data on demographics, health conditions, disease‐specific clinical characteristics, and perception of disease‐specific stigma. Participants were recruited from a large internet panel (Kantar Profiles) via a quota‐sampling approach to mirror the US general adult population [8]. Adults who reported experiencing AA and receiving a physician diagnosis of AA were included.

### 2.2 Outcomes

Prevalence of self‐reported physician‐diagnosed AA was calculated. Respondents' sociodemographic and comorbidity profile, AA clinical characteristics, and perception of stigma were analyzed overall and stratified by self‐reported disease severity (mild, moderate, and severe). Survey respondents were not provided further definitions of mild, moderate, or severe AA, and responses were based on an interpretation of their own disease severity. Respondents conveyed perceptions of stigma by rating their experience with feelings of embarrassment, negative judgment, and being treated negatively due to AA on a Likert‐type, 3‐item, composite scale, with each item scored from 1 (not at all) to 7 (very much so).

### 2.3 Statistical Analysis

Data were weighted based on age, race/ethnicity, sex, and education to match the demographic composition of the US adult population based on the 2022 Current Population Survey of the US Census Bureau [8]. Results were reported as weighted totals and percentages. Frequencies and percentages were reported for categorical variables, with means and standard deviations (SDs) for continuous or discrete variables. Analyses were performed using Unix Quantum Version 5.8.1 (Mission Hills, CA).

## 3 Results

### 3.1 Respondents

An unweighted sample of 75 007 adults completed the survey (weighted *n* = 255.7 million; Figure S1), of which 859 (weighted *n* = 2.9 million) reported a clinical diagnosis of AA (1.1% of the adult population). Of those with a clinical AA diagnosis, 58.8%, 26.2%, and 15.0% rated their AA as mild, moderate, and severe, respectively.

Mean age was 48.6 years and 57.2% were female (Table 1). At the time of the survey, 41.9% of respondents were experiencing hair loss (Table 2). Overall, 37.1% of respondents were receiving medications for AA. Respondents with severe disease reported higher use of oral treatments (including Janus kinase inhibition) and injectable treatments.

**TABLE 1:** Demographics and baseline characteristics (weighted, *n* represented in thousands).

Characteristic	Diagnosed with AA^a^			
	Total (*N* = 2862)	Mild (*n* = 1684)	Moderate (*n* = 749)	Severe (*n* = 430)
Age, mean (SD)	48.6 (16.7)	48.0 (17.0)	48.0 (17.0)	51.9 (16.1)
Sex, *n* (%)				
Female	1636 (57.2)	898 (53.3)	476 (63.6)	262 (61.1)
Male	1226 (42.8)	786 (46.7)	273 (36.4)	167 (38.9)
Race/ethnicity, *n* (%)				
American Indian	8 (0.3)	5 (0.3)	3 (0.4)	0
Asian	145 (5.1)	103 (6.1)	30 (4.0)	12 (2.9)
Black or African American	548 (19.1)	250 (14.8)	172 (23.0)	126 (29.3)
Hispanic	629 (22.0)	404 (24.0)	147 (19.6)	79 (18.4)
Mixed	63 (2.2)	35 (2.1)	17 (2.3)	11 (2.5)
White	1416 (49.5)	861 (51.1)	361 (48.2)	194 (45.0)
Other	54 (1.9)	26 (1.6)	19 (2.6)	8 (1.9)
Region, *n* (%)				
South	1122 (39.2)	641 (38.1)	266 (35.5)	215 (50.1)
Northeast	679 (23.7)	367 (21.8)	213 (28.5)	99 (23.1)
West	602 (21.0)	386 (22.9)	146 (19.5)	71 (16.4)
Midwest	459 (16.0)	291 (17.3)	124 (16.5)	45 (10.4)
Education, *n* (%)				
Less than university	1703 (59.5)	979 (58.1)	465 (62.2)	259 (60.2)
University or higher	1156 (40.4)	702 (41.7)	283 (37.8)	171 (39.8)
Insurance coverage, *n* (%)				
Yes	2539 (88.7)	1507 (89.5)	637 (85.1)	395 (92.0)
No	324 (11.3)	177 (10.5)	112 (14.9)	34 (8.0)
Marital status, *n* (%)				
Married	1571 (54.9)	1001 (59.4)	373 (49.8)	197 (46.0)
Single, never married	594 (20.7)	301 (17.9)	164 (21.9)	128 (29.8)
Divorced	348 (12.2)	163 (9.7)	114 (15.2)	71 (16.5)
Widowed	158 (5.5)	92 (5.5)	49 (6.5)	18 (4.1)
Living with partner	124 (4.3)	81 (4.8)	39 (5.3)	4 (0.9)
Separated	51 (1.8)	35 (2.1)	6 (0.8)	10 (2.3)
Decline to answer	16 (0.6)	11 (0.6)	3 (0.4)	2 (0.5)

Abbreviations: AA, alopecia areata; SD, standard deviation.

^a^ Data weighted based on age, race/ethnicity, sex, and education to match the demographic composition of the US adult population. The unweighted totals for total respondents and those reporting mild, moderate, or severe AA are 859, 512, 222, and 125 responders, respectively.

**TABLE 2:** Clinical characteristics (weighted, *n* represented in thousands).

Characteristic, *n* (%)	Diagnosed with AA^a^			
	Total (*N* = 2862)	Mild (*n* = 1684)	Moderate (*n* = 749)	Severe (*n* = 430)
Diagnosing physician	*n* = 2862	*n* = 1684	*n* = 749	*n* = 430
Dermatologist	1311 (45.8)	767 (45.6)	304 (40.6)	240 (55.9)
Primary care physician/GP/internist	760 (26.6)	456 (27.1)	190 (25.4)	113 (26.4)
Unknown/not reported	365 (12.8)	209 (12.4)	112 (14.9)	45 (10.5)
NP/PA	225 (7.9)	139 (8.2)	72 (9.6)	15 (3.4)
Other	144 (5.0)	92 (5.4)	40 (5.4)	12 (2.9)
Pediatrician	57 (2.0)	22 (1.3)	31 (4.2)	4 (0.9)
Time since first diagnosis, mean (SD), years	16.1 (15.4)	16.6 (16.0)	13.7 (14.4)	18.2 (14.6)
Currently experiencing an episode of AA	*N* = 2862	*n* = 1684	*n* = 749	*n* = 430
No	1069 (37.3)	784 (46.5)	199 (26.6)	86 (20.0)
Yes, but not experiencing hair loss due to treatment	594 (20.7)	397 (23.6)	159 (21.3)	38 (8.8)
Yes, currently experiencing hair loss	1200 (41.9)	504 (29.9)	390 (52.1)	306 (71.3)
Areas of hair loss in current episode	*N* = 1200	*n* = 504	*n* = 390	*n* = 306
Scalp	902 (75.2)	324 (64.3)	299 (76.6)	280 (91.3)
Eyebrows	410 (34.1)	161 (32.0)	141 (36.1)	108 (35.1)
Eyelashes	256 (21.3)	102 (20.3)	65 (16.7)	88 (28.8)
Beard	216 (18.0)	83 (16.5)	64 (16.3)	69 (22.5)
Body	173 (14.5)	35 (7.0)	55 (14.1)	83 (27.2)
Mustache	107 (8.9)	46 (9.2)	16 (4.0)	45 (14.6)
Currently receiving treatment for AA	*N* = 2862	*n* = 1684	*n* = 749	*n* = 430
Yes	1063 (37.1)	653 (38.8)	281 (37.6)	129 (29.9)
Current AA treatment^b^	*N* = 1063	*n* = 653	*n* = 281	*n* = 129
Topical^c^	594 (55.9)	391 (59.8)	135 (48.0)	68 (53.0)
Oral	489 (46.0)	290 (44.3)	141 (50.3)	58 (45.0)
Oral immunosuppressants	156 (31.8)	90 (31.1)	45 (32.1)	20 (34.9)
Oral corticosteroids	139 (28.4)	84 (29.0)	53 (37.7)	2 (2.8)
Oral antihistamines	94 (19.2)	69 (23.9)	19 (13.2)	6 (10.4)
Other oral treatment	85 (17.4)	48 (16.6)	28 (19.5)	9 (16.1)
Oral minoxidil	51 (10.3)	37 (12.9)	12 (8.1)	2 (2.9)
Oral treatment including JAK inhibition^d^	17 (3.5)	3 (1.0)	3 (2.4)	11 (18.8)
Unknown^e^	70 (14.4)	39 (13.6)	19 (13.2)	12 (21.2)
Injection at areas of hair loss	273 (25.7)	165 (25.2)	58 (20.5)	51 (39.3)
Other injectable	202 (19.1)	115 (17.7)	47 (16.6)	40 (31.4)
Other	117 (11.1)	45 (6.9)	56 (20.0)	16 (12.7)
None listed	61 (5.8)	35 (5.4)	16 (5.7)	10 (7.9)
Mental health and sleep disturbances in the past 12 months	*N* = 2862	*n* = 1684	*n* = 749	*n* = 430
Emotional or mental health^f^	1147 (40.1)	617 (36.6)	360 (48.1)	171 |(39.7)
Anxiety^g^	835 (29.2)	452 (26.8)	232 (31.0)	151 (35.1)
Depression	702 (24.5)	376 (22.4)	208 (27.8)	118 (27.4)
PTSD	252 (8.8)	115 (6.8)	98 (13.1)	39 (9.2)
Sleep conditions^h^	737 (25.7)	399 (23.7)	215 (28.7)	123 (28.6)
Insomnia	396 (13.8)	199 (11.8)	119 (15.9)	78 (18.1)
Sleep difficulties^i^	205 (7.2)	106 (6.3)	65 (8.7)	35 (8.1)
Psychological counseling for anxiety, depression, or sleep disorder related to AA	310 (10.8)	161 (9.6)	111 (14.9)	37 (8.7)
Prescribed medication for treatment of anxiety, depression, or sleep disorder related to AA	309 (10.8)	126 (7.5)	131 (17.5)	52 (12.0)

Abbreviations: AA, alopecia areata; GP, general practitioner; JAK, Janus kinase; NP, nurse practitioner; PA, physician assistant; PTSD, post‐traumatic stress disorder; SD, standard deviation.

^a^ Data weighted based on age, race/ethnicity, sex, and education to match the demographic composition of the US adult population. The unweighted totals for total respondents and those diagnosed with mild, moderate, or severe AA are 859, 512, 222, and 125 responders, respectively.

^b^ Current AA treatment routes of administration are not mutually exclusive.

^c^ Topical therapy included corticosteroids and other treatments.

^d^ Includes baricitinib and ruxolitinib.

^e^ Patient selected taking oral therapy but did not select any oral medications.

^f^ Emotional or mental health conditions included attention deficit/deficit and hyperactivity disorder, obsessive compulsive disorder, panic disorder, and phobias. Anxiety, depression, and PTSD are not mutually exclusive.

^g^ Includes anxiety, generalized anxiety disorder, or social anxiety disorder.

^h^ Sleep conditions included insomnia, sleep difficulties, narcolepsy, sleep apnea, and idiopathic hypersomnia.

^i^ Other than insomnia, narcolepsy, or sleep apnea.

Overall, 40.1% and 25.7% of respondents reported experiencing symptoms of a diagnosed emotional/mental health condition or a sleep condition, respectively, in the past 12 months (Table 2). A total of 10.8% of respondents were receiving psychological counseling, and 10.8% were receiving medications for AA‐related anxiety, depression, or sleep disorders.

### 3.2 Respondent Satisfaction With Current Hair Growth

**FIGURE 1:**
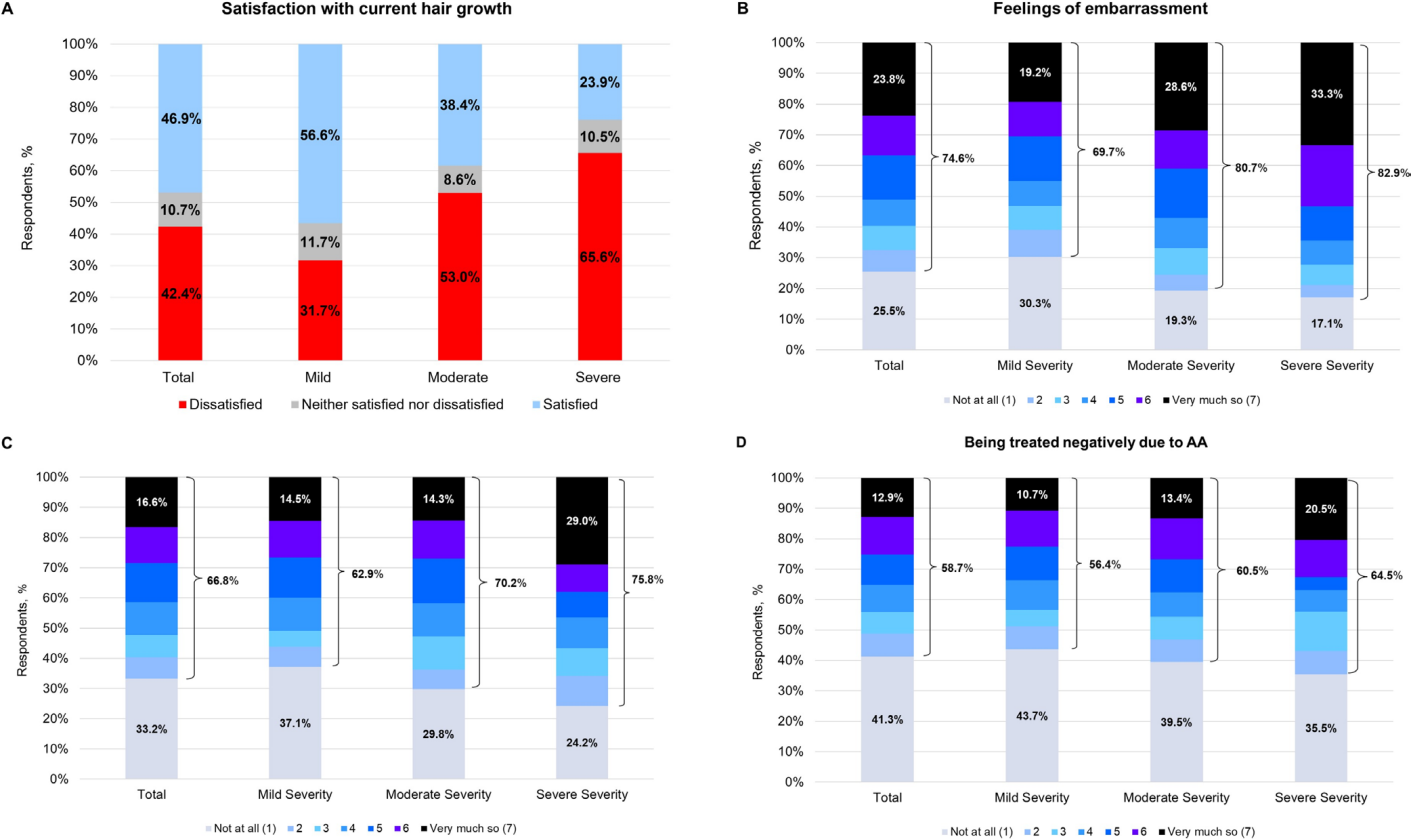
(A) Satisfaction with current hair growth among respondents and perceived stigma among respondents with (B) feelings of embarrassment, (C) negative judgment, and (D) being treated negatively due to AA. AA, alopecia areata.

Overall, 42.4% of respondents were dissatisfied with their current hair growth (Figure 1A). Dissatisfaction increased with worsening disease severity; 31.7%, 53.0%, and 65.6% of respondents with mild, moderate, or severe AA, respectively, were dissatisfied with their hair growth.

### 3.3 Perceived Stigma Among Adults With AA

Stigma was reported by 79.2% of respondents. The rate of perceived stigma increased with worsening disease severity (Figure 1B–D). Overall, 74.6% felt embarrassed due to their AA (Figure 1B), 66.8% reported feeling judged negatively by others because of their AA (Figure 1C), and 58.7% felt negatively treated due to their AA (Figure 1D). In total, 54.8% of patients reported any level of perceived stigma (2–7 on scale) compared with 20.8% who reported not at all (1 on scale) to all three stigma questions.

## 4 Discussion

The prevalence of AA in adults in our study (2.9 million [1.1%]) aligns with published estimates of 1.5% [9]. In this study, a higher proportion of patients with AA had depressive/anxiety disorders than general population estimates [10, 11]. One‐quarter of respondents reported physician‐diagnosed depression versus 1 in 5 US adults with a self‐reported depression diagnosis [10]. A systematic review of observational and interventional studies showed that 7% to 17% of patients with AA have depressive or anxiety disorders requiring psychiatric care [11]. Despite 40% of respondents experiencing symptoms of diagnosed emotional/mental health conditions and ~25% experiencing symptoms of diagnosed sleep conditions, only ~10% were receiving counseling or therapeutic intervention for these disorders.

Approximately one‐third of respondents were receiving treatment for AA. The most common routes of administration were topical or oral. Topical therapy, typically corticosteroids, is often an initial treatment strategy for patchy AA and mild scalp hair loss [12], while systemic therapy is generally recommended for patients with more extensive hair loss [13]. Only a small proportion of respondents reported using Janus kinase inhibitors. As the satisfaction with current hair growth in respondents with AA was low, our results suggest unmet treatment needs. The recent availability of therapies for severe AA provides an opportunity for more effective AA management.

Respondents reported substantial internalized and external stigma across all levels of AA severity, with increasing magnitude reported with worsening disease severity, highlighting the impact of external perceptions of AA on adults. Our findings are consistent with published data, which suggest that a large proportion of patients with AA experience stigma and decreased quality of life [3, 4, 14] and stigmatizing layperson attitudes toward individuals with AA exist across multiple social/professional scenarios [15]. These stigmas may lead to increased social withdrawal and/or increased feelings of isolation in individuals with AA, further exacerbating psychosocial burdens or mental health.

Our results should be interpreted in the context of some limitations. The NHWS uses self‐reported data; clinical verification of responses was not possible. Recall error or other response biases may exist given the nature of the data, and survey respondents were not provided further definitions of mild, moderate, or severe. Although this intentional decision to omit any definitions may introduce variability in the measurement, it allowed respondents to reflect upon their own interpretation of disease severity, which may be influenced by a number of individual factors, such as the extent of disease, the location of hair loss, or the impact on work/school and/or quality of life. Although the NHWS is designed to reflect the demographic composition of the general US population, our results may not be generalizable to the entire AA population as patients < 18 years of age were not included in this study. No active measures were taken to eliminate responder participation bias. However, the NHWS starts as a general population survey with additional questions about AA triggered only after the patient‐reported experience with or diagnosis of AA; the survey was weighted to the demographic distribution of the adult US population to address potential differences across social groups that may be skewed based on participation rates. Additionally, reporting only diagnosed emotional/mental health conditions may have resulted in underestimation due to stigmatization/underutilization or lack of access to mental healthcare services. While identifying the differences in the comorbidities of AA by race was not within the scope of this study, we believe this is an important next step in future studies to assess clinical burden across subpopulations.

Adults with AA reported substantial emotional/mental health comorbidities, dissatisfaction with their current hair growth, and AA‐related stigma. These findings suggest that unmet treatment needs exist, particularly among patients with more severe AA. Further research is warranted to understand how emerging AA treatments may improve stigma and whether the degree of stigma improvement is affected by treatment results.

## Ethics Statement

Institutional review board approval was not required for this study; however, the protocols and materials associated with the 2023 US NHWS original fielding were reviewed by Pearl Institute Review Board (Indianapolis, IN) and granted exemption from expedited or full ethical review. Respondents provided informed consent.

## Conflicts of Interest

G.G., E.H.L., H.T., L.N., G.B., M.S., and S.K.K. are employees of and own stock and/or stock options in Pfizer. L.L. and N.S. are employees of Oracle Life Sciences. K.A.H. was a consultant for Pfizer at the time of this analysis.

## Supporting information


Data S1.

## Data Availability

The data that support the findings of this study are available from Oracle Life Sciences, but restrictions apply to the availability of these data, which were used under license for the current study, and so are not publicly available.
