# Supplementary material for: Perceived Stigma Among Adults With Alopecia Areata in the United States
Source: J Dermatol. 2025 May 23;52(7):1185–91. doi: 10.1111/1346-8138.17786 (PMC12231919; doi:10.1111/1346-8138.17786)
Supplement: Supplementary file 1 — Data S1. [file JDE-52-1185-s001.docx]

**Figure S1.** Responder flow diagram


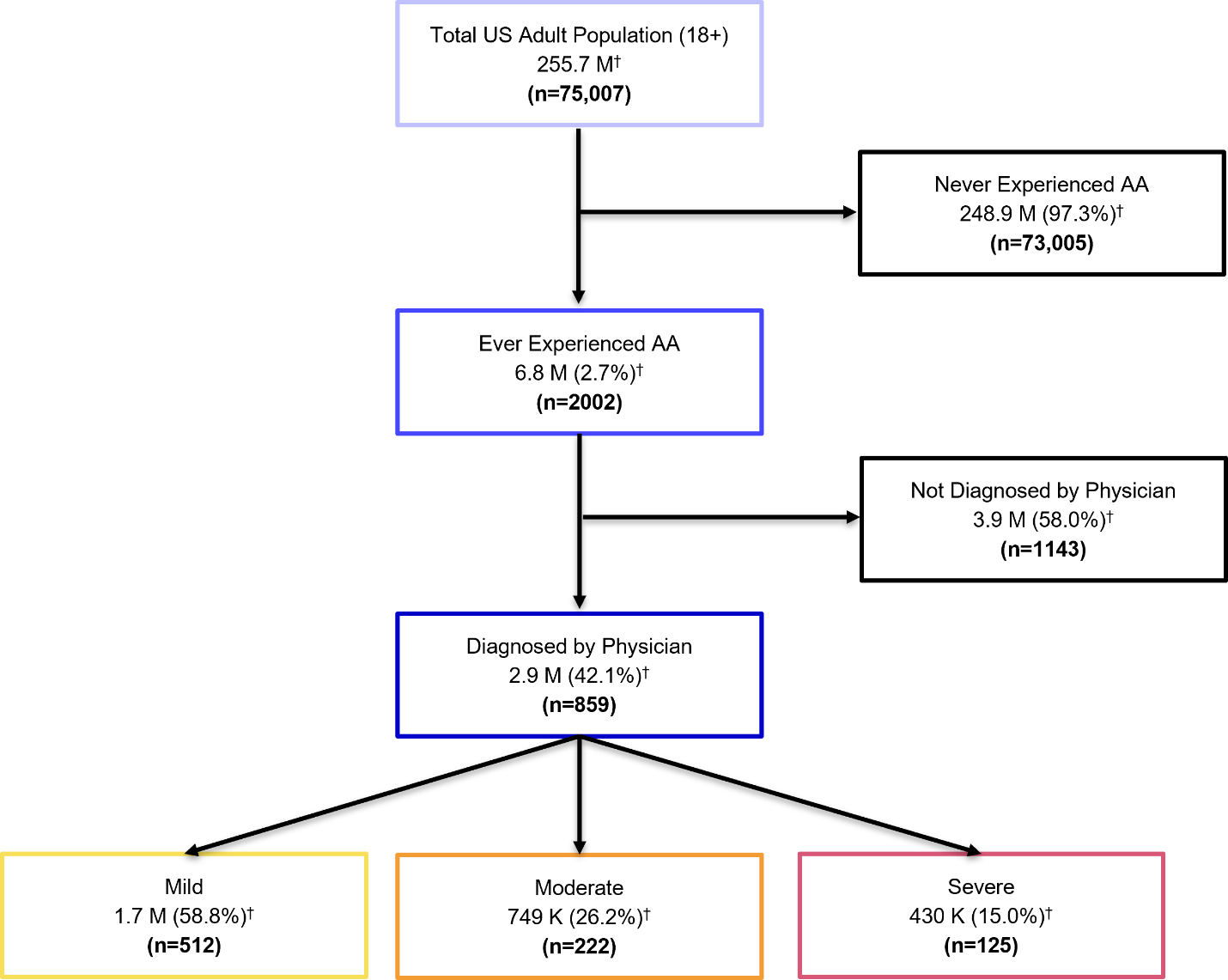


AA, alopecia areata; K, thousand; M, million.

^†^Weighted to the US census population.
